# Promotion and COVID-19 lockdown increase uptake of funded maternal pertussis vaccination in pharmacy: A mixed methods study

**DOI:** 10.1371/journal.pone.0307971

**Published:** 2024-08-29

**Authors:** Natalie J. Gauld, Cath Knapton, Owen Sinclair, Cameron C. Grant

**Affiliations:** 1 Department of Paediatrics: Child and Youth Health, University of Auckland, Auckland, New Zealand; 2 School of Pharmacy, University of Auckland, Auckland, New Zealand; 3 Mid-Central Community Pharmacy Group, Hamilton, New Zealand; 4 Paediatrics, Te Whatu Ora Waitematā, Auckland, New Zealand; King Faisal University, SAUDI ARABIA

## Abstract

Pertussis vaccination is recommended during pregnancy to protect the baby. Pertussis vaccination was initially free to pregnant people through general practice and hospitals in New Zealand, but uptake was suboptimal. In one district funding of maternal pertussis vaccination was widened to community pharmacies in 2016. Eighteen months later promotion to pharmacies, midwives and pregnant people took place. In 2020 and 2021, COVID-19 lockdowns occurred. Aim. To explore the effects of promotion and COVID-19 lockdowns on uptake of funded maternal pertussis vaccination in pharmacy, and awareness, use and opinions of promotional elements. Methods. Five years of pharmacy claims data were analysed and 12 pharmacists, 18 people eligible/recently eligible for maternal pertussis vaccination and 11 midwives were interviewed. Results. Provision of maternal pertussis vaccination increased during and after promotion. Qualitative data showed that pharmacists valued phone calls with information about maternal pertussis vaccination and recommendations for increasing uptake. Prompted by these calls, some pharmacists contacted midwives to inform them of funded maternal pertussis vaccination in the pharmacy (which midwives appreciated) and recommended pertussis vaccination to pregnant clients. Pharmacy staff reportedly were motivated to recommend this vaccination by being informed about it and having posters displayed in the pharmacy. Pregnant people valued healthcare professionals’ conversations about maternal pertussis vaccination, but appeared to be uninfluenced by posters and promotional social media posts about this vaccination. During COVID-19, maternal pertussis uptake in pharmacies increased 31% March to May 2020 (before and during the first COVID-19 lockdown) versus the same time the previous year, then declined. Conclusion. Promotion appeared to have a sustained effect on uptake of maternal pertussis vaccination in pharmacies. Pregnant people were most influenced by discussions with healthcare professionals. Pharmacists and pharmacy staff increased proactivity with maternal vaccinations after promotion to them. Promotion may need to be repeated over time.

## Introduction

Community pharmacy is increasingly being used to aid access to vaccinations, with extended hours of opening, walk-in services, and potential to provide vaccines opportunistically [1,2]. Uptake of vaccinations has increased through community pharmacists providing education about and/or administering vaccines, but often any increase in uptake has been modest [3–5], and sometimes not sustained [4,6].

As pertussis (whooping cough) causes hospitalisations and deaths, particularly of young infants [7,8], maternal pertussis vaccinations (MPV) are recommended in every pregnancy to protect the infant [9]. However, uptake is suboptimal in many countries, including New Zealand [10–13], particularly in population groups experiencing greater socioeconomic deprivation [11,14], and in indigenous people [11,13]. In New Zealand, this low uptake has occurred despite being fully funded (free to the patient) through general practices and hospitals since 2013.

Reasons why pregnant people do not receive maternal vaccinations include lack of awareness, misperceptions, safety concerns, difficulty accessing vaccinations, needle phobia, and other priorities [15–22]. While healthcare professional (HCP) recommendation and/or endorsement of safety of the vaccination encourages uptake [10,12,15,16,20,22], sometimes antenatal care providers are not informing people about MPV [10,16,22]. Uptake of MPV can be increased by working with vaccination providers, while randomised controlled trials of educational strategies with pregnant people did not find increased uptake[23]. Provision by midwives at antenatal care is particularly effective at increasing MPV uptake [23], but this is logistically difficult for most midwives in New Zealand [22], so alternative access needs to be optimised. It has been recommended that funding for MPV is extended to community pharmacies to help address barriers to access including low awareness [16].

Research related to this manuscript showed that funded availability of maternal vaccinations through community pharmacies can help awareness and access [24]. Uptake of MPV increased significantly more in the intervention region, where funded MPV (free for the patient) was available in pharmacies, general practice and hospitals, than in control regions, where MPV was only funded in general practice and hospitals but not in pharmacies [5]. This paper examines the effect of promotion and COVID-19 lockdowns in the community on the uptake of MPV through pharmacy, and reports information from the qualitative interviews with those eligible or recently eligible (pregnant people or person who had a baby in the last 12 months) for the service and HCPs on the promotional materials used.

Our aim was to explore how uptake of funded MPV through pharmacy is affected by promotion and COVID-19 lockdowns and the COVID-19 pandemic in New Zealand.

The objectives were to: (1) measure uptake of MPV in pharmacy before, during and after a six-month promotional period; (2) explore which promotional elements were perceived to aid uptake and recommendation of MPV; and (3) compare uptake of MPV in pharmacy during the COVID-19 pandemic including lockdowns with data from the same period in two previous years.

With five years of data and qualitative data, we provided insight on the effect of promotion and the COVID-19 pandemic and lockdowns on uptake of MPV in pharmacy in New Zealand.

## Materials and methods

Ethics Committee approval was granted by the New Zealand Ministry of Health Northern B Health and Disability Ethics Committee (18/NTB/43). Written informed consent was obtained for all interviews, collected at the time of the interview (face-to-face) or received by email from the participant for telephone interviews.

### Setting

The study was conducted in the region defined by the Waikato District Health Board. Approximately 458,000 people reside in the region, in which is situated a city of 165,000. There are 5,200–5,500 births per year in this region [25].

MPV has been government-funded in New Zealand from general practice (since 2013), and hospitals with consumer information leaflets available. Pharmacists could administer unfunded pertussis vaccination from 2013. We conducted a research project of an intervention in which funding for MPV was extended to community pharmacies in Waikato from 1 November 2016. This meant MPV could be administered at no cost to the patient from pharmacy in addition to general practice and hospitals. This research measured uptake and conducted interviews to understand experiences, opinions and behaviour regarding MPV including its funded availability in pharmacies.

There are 83 community pharmacies in Waikato, and of these, 35 (42%) provided vaccination services as at April 2018 [5].

### Promotion

As part of the study, promotion of MPV in the Waikato district took place from April 2018 to October 2018 (Panel 1).

**Panel 1. Promotional activity undertaken with pharmacies and midwives**

Promotional activity undertaken with pharmacies as part of the research project

    • A free evening training meeting covering maternal vaccinations for pharmacists in the main city in Waikato in October 2016.

    • All other promotional activity took place from mid-April 2018 to mid-October 2018.

    • Two phone calls to the lead vaccinator in each pharmacy or the pharmacist in non-vaccinating pharmacies in April 2018 and June 2018

        ○ Asking if the pharmacy provided vaccinations or not.

If not providing vaccinations, encouragement to do so.

        ○ Reminding them of the importance of maternal vaccinations.

        ○ Suggesting they contact their local midwives to advise they provided free maternal vaccinations (if the pharmacy provided the vaccination service).

        ○ Recommending maternal vaccinations when people came into the pharmacy for supplements recommended during pregnancy (iodine or folic acid)

        ○ Reminding pharmacists to enter pertussis vaccinations on the         ○ National Immunisation Register.

        ○ Collecting email addresses for pharmacies with social media pages to allow links to be sent to them for use on social media.

• Posters were provided to all pharmacies to display about MPV including availability in pharmacy and general practice.

• An A4 laminated Fact Sheet with key points on maternal pertussis and influenza vaccinations was provided to all pharmacies with the suggestion that it be put on the wall in the pharmacy staffroom.

• Two quizzes aimed at frontline pharmacy staff about maternal vaccinations were faxed to all pharmacies, along with the same Fact Sheet, with a prize draw (five prizes of $50 gift voucher in each case) for the correct answers.

• Twelve social media posts promoting MPV were put on Facebook, with a target audience of female consumers aged 20–55 years and later 15–65 years who used in their Facebook posts keywords related to pregnancy. These posts included three brief videos promoting maternal vaccinations and noting their availability from pharmacies (intervention area for maternal pertussis and influenza, and control area for maternal influenza) and general practice (all areas). One of the videos was translated into Te Reo (language of New Zealand’s indigenous Māori). One was animated and intended to be humorous, a second was a Māori paediatrician providing vaccination advice for pregnant people, and a third included a video of a baby hospitalised with pertussis. All advised maternal vaccinations and explained they were free from pharmacy and general practice.

• Vaccinating pharmacies with social media pages were emailed a link to social media.

• Free t-shirts promoting MPV were offered to pharmacy team members.

• Emails were sent via the College of Midwives to their members with the following messages:

        ○ Notifying that pharmacy could administer free maternal influenza and pertussis vaccinations.

        ○ Reminding that maternal influenza and pertussis vaccinations are also free in general practice.

        ○ Providing brief key points about maternal vaccinations.

        ○ Offering posters if desired.

        ○ Providing a list of pharmacies in the region which provided vaccinations.

    • A 3-minute maternal vaccination update video for HCPs was shared via email via a link to pharmacies for which we had email addresses and was sent by the Pharmaceutical Society of New Zealand to their pharmacist members in the Waikato region (virtually all pharmacists at that time). This video was also shared with midwives through the College of Midwives local branch contacts who were asked to email the link to their members with a reminder of maternal vaccinations being funded through pharmacies and general practice.

    • Given the importance of addressing inequity in uptake of maternal vaccinations, promotional activity had input from the Māori leader of the Hapu Wananga programme (an antenatal programme for pregnant people who were Māori) at Waikato District Health Board, and Dr Owen Sinclair (Paediatrician whose Māori affiliations are Iwi Te Rarawa, Hapu as Ngati Manu/Ngati Pahuwera).

Separate to the promotion regarding this research, pamphlets about influenza vaccination and pertussis vaccination in pregnancy were also available in general practice, through midwives, and through pharmacy (from late 2016). Only the pharmacy pamphlets mentioned pharmacy availability of funded MPV.

### COVID-19 in New Zealand

The first confirmed New Zealand case of COVID-19 was on 28 February 2020, with nationwide lockdown from 26 March. This level four lockdown saw non-essential business closed to staff and the public, schooling on-line only, no gatherings, no mixing households, and many recreational activities banned. Only essential businesses could open e.g., supermarkets, pharmacies and petrol stations. One month later level three lockdown commenced, allowing mixing with one other household, up to 10 people for specific events and restricted work and school attendance. By 22 May 2020, there were no new cases of COVID-19 transmitted in the community and from 10 June all of New Zealand was under level one with no restrictions in domestic travel, events or school attendance. Waikato remained COVID-19-free until a single case in February 2021. The delta outbreak started August 2021 and saw a return to level four lockdown nationwide, this eased in Waikato soon after although localised areas of Waikato had cases and stricter lockdowns at times. High levels of COVID-19 vaccination saw a move from elimination to suppression on 4 October 2021. See S1 Table for a COVID-19 timeline for New Zealand and lockdown details.

During the first lockdown general practices swiftly moved to telehealth for most consultations, with few in-person visits [26].

### Quantitative data sources and analysis

Quantitative data were extracted from the de-identified claims excel spreadsheet from the Midlands Community Pharmacy Group from the pharmacy manual invoicing for funded MPV from 1 November 2016 through to October 2021 (data provided 8 December 2021). This included data from all Waikato pharmacies administering and claiming a fee for MPV during this time, including the date the MPV was given. Data were extracted by month of administration and then analysed by six-month periods. This analysis included graphing the results and calculating percentage change by six-month period compared to the last six-month period. For the COVID-19 pandemic period monthly administration was used and compared to the equivalent period the previous year in a graph. Differences were calculated and reported as a percentage where appropriate.

### Qualitative interviews

Qualitative data were taken from 41 interviews of consumers, pharmacists and midwives. Methods and results from these interviews have been reported elsewhere, but are outlined below [22,24,27].

Consumers (people who were pregnant or who had had a baby in the last 12 months) were invited to participate from recruitment in a pharmacy or by a midwife with a view to maximum variation [28] (e.g., in parity, age, location, and maternal vaccinations received). For ethnicity, we targeted having half of consumer participants to be Māori, and one-third to half of midwives to be Māori. Pharmacists and midwives were purposively selected for a variety of experience and locations, but particularly including lower socio-economic areas and rural communities. Community pharmacies providing vaccinations were selected by NG, telephoned, and the pharmacist who administered most vaccinations in the pharmacy was invited to participate. To understand the knowledge about maternal vaccinations and use of promotional materials for maternal vaccinations by pharmacists in non-vaccinating pharmacies, owners of two non-vaccinating rural pharmacies serving populations that included lower socio-economic areas were invited to be interviewed. Midwives were selected with the help of College of Midwives aiming for maximum variation [28] (e.g. variety of ages, locations and ethnicities), but with most serving a lower socio-economic area. One midwife was approached for an interview after suggestion as an information-rich source from another midwife who was interviewed (snowballing [28]). The midwives were contacted by email or telephone and invited to participate in an interview.

Half of the consumers were recruited and interviewed face-to-face in a private consulting room in two pharmacies (rural and city) by a male Māori pharmacist who had received training on interviewing. All other participants were recruited and interviewed face-to-face (n = 26) or by telephone (n = 6) by NG, a female New Zealand European pharmacist and researcher, experienced in interviewing. Both interviewers have positive views about vaccination.

Using an implementation science approach [29,30], interviews were semi-structured using a conversational approach, dynamic in being able to probe areas arising in the interview, and able to change questions according to the earlier responses, and time-conscious, exploring some areas further where time permitted.

Interviews for consumers included questions relevant to this paper about the patient journey including information sources about maternal vaccinations and influences on their maternal vaccination decisions, probing whether they had seen posters, pamphlets, Facebook posts, and their views on these elements. They were asked about discussion about the vaccinations with HCPs and asked “What could help other women like you to find out about the vaccination?”. Demographics and maternal vaccination use were collected.

For pharmacists and midwives, questions relevant to this research included: what helps you provide vaccinations in your practice/pharmacy including probes on use and opinions of resources (including promotional material), and about communication between the pharmacy and midwives on free maternal vaccinations in pharmacy. They were asked if they had seen or used the Facebook maternal vaccination posts (pharmacy were sent these and could share on their page), and their opinions and experiences with these. Some were shown the videos about maternal vaccinations used as promotion on Facebook by this project for their views. They were also asked what needs to be done to increase the number of women getting vaccinations in pregnancy. Demographic data were collected and, for vaccinating pharmacists, the number of MPVs provided. Midwives were asked about awareness of free maternal vaccinations in pharmacy and source of that awareness.

After obtaining informed consent, interviews were conducted in person or by telephone, then a koha (gift) of a NZ$30 gift voucher was given. Interviews were audio-recorded then transcribed verbatim and checked against the recording (n = 40), or notes were taken without recording if preferred by the participant (n = 1). Transcripts or notes were entered into Nvivo 12 Pro and were coded then analysed systematically node by node (QSR International) by NG. The results were shared amongst all researchers for comment. One node included views and use of promotional material, which is presented here.

Analysis related to this paper was pragmatic qualitative analysis for implementation science [30] aiming to explain use and views of promotional material (by consumers and HCPs), information sources for consumers about maternal vaccinations, and recommendations for how to increase uptake of maternal vaccinations.

## Results

### Quantitative results

Pharmacy claims for 3,423 MPVs were used for the period 1 November 2016 to 31 October 2021.

**Fig 1:**
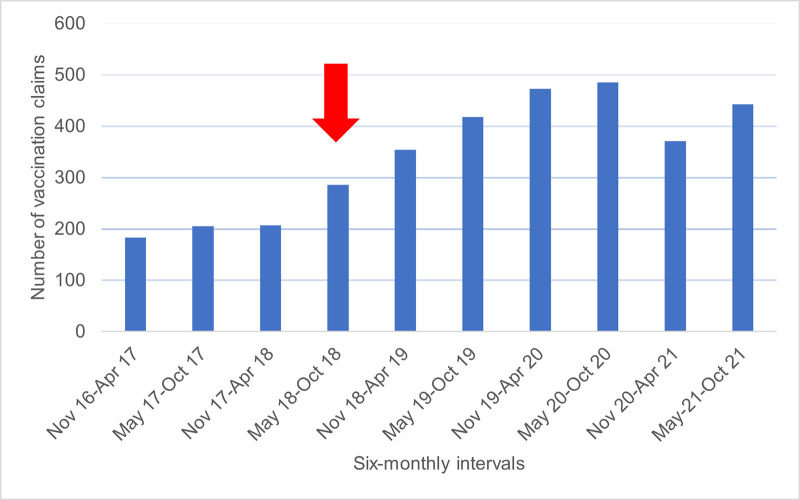
Five years of funded maternal pertussis vaccinations in pharmacy before, during and after promotion and Covid-19 by six-monthly intervals.

During the five years of funding the MPV in pharmacy considerable change occurred in vaccine uptake (Fig 1). For the first 18 months of the programme, before promotion started, vaccine uptake was flat, averaging 33 MPV per month total across vaccinating pharmacies. Uptake of MPV during the six-month period of promotion increased by 38% on the previous six months, averaging 48 MPV per month. Further growth occurred over the following two years. In the three six-month periods following the promotional activity MPV uptake increased 24%, 18%, and then 13%. In the 12-months after the promotional period, an average of 64 MPV administrations per month were claimed, a 94% increase on the pre-promotional period. The peak period occurred in May to October 2020 with 81 MPV administrations per month across all pharmacies.

Following this peak, a 24% decline in pharmacy administration occurred in the November 2020-April 2021 period, followed by a 20% increase in the May-October 2021 period, with the final period of uptake below the earlier peak.

For the calendar year 2020, pharmacy administration of vaccines (n = 939) reached approximately 17–18% of pregnant people based on 5,200–5,500 births per year in Waikato during this time.

[Fig 1 footnote immediately below the figure:] The red arrow represents the six-month period of promotion which started mid-April 2018 and continued to mid-October 2018.

For context for Fig 1, to understand the COVID-19 effects in NZ during 2020, the first COVID-19 case in New Zealand was announced on the 28^th^ of February 2020. By 14 March 2020 new cases were being announced daily. The first Covid-19 lockdown in New Zealand occurred from 26 March to 14 May 2020 (the strictest levels 4 and 3; see S1 Table for explanations). COVID-19 infections had been eliminated from the community by 29 May 2020 and the lockdown lifted 10 June 2020.

**Fig 2:**
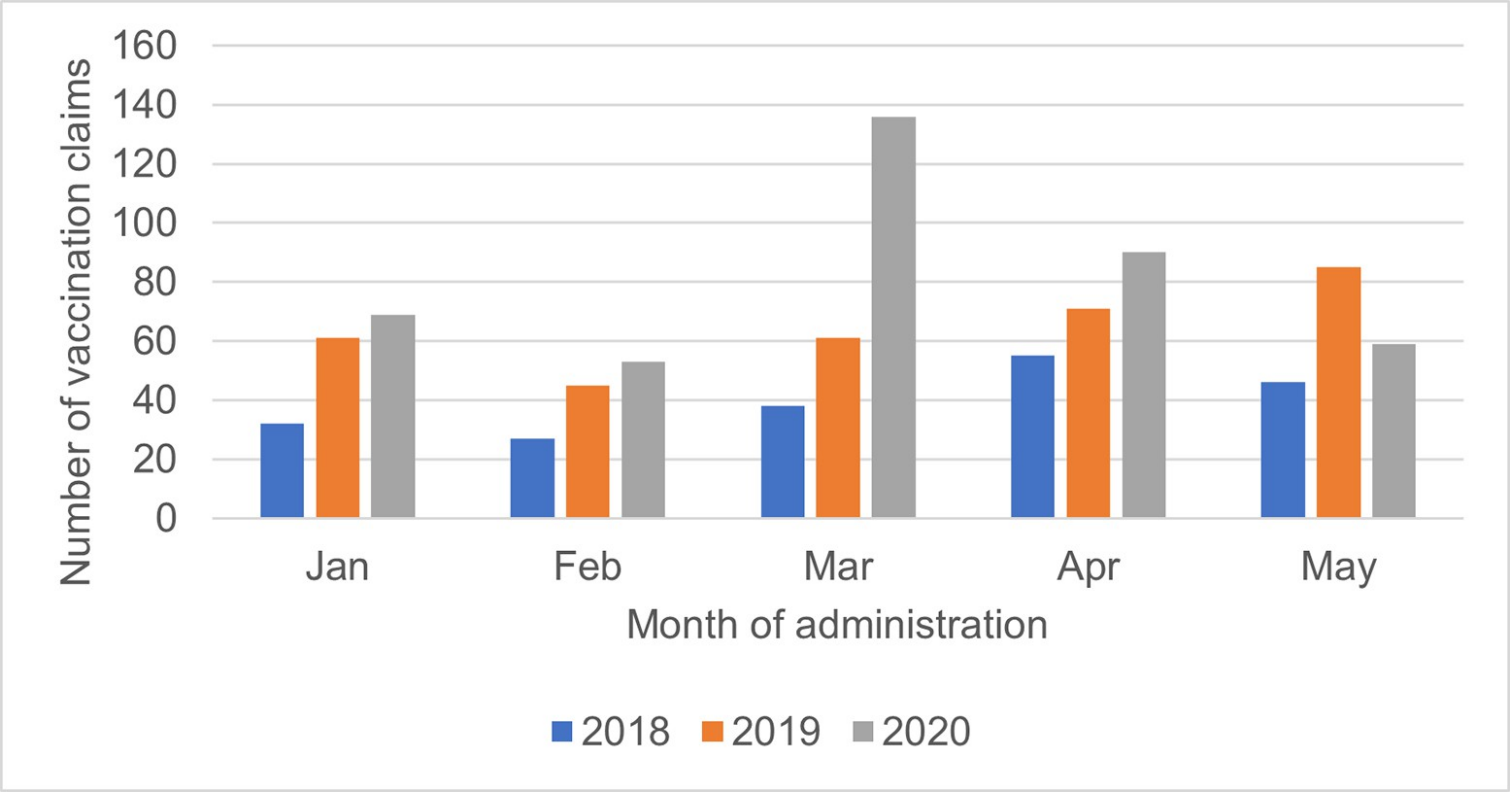
Pertussis vaccination uptake in the first COVID-19 Lockdown.

MPV uptake in pharmacy increased as COVID-19 cases increased in the community and the lockdown started (Fig 2). In March 2020 pharmacy MPV administrations were 122% higher than March 2019 (Fig 2). From March to May 2020 a time of increasing COVID-19 cases in the community and the first and very restrictive lockdown, MPV administrations in pharmacies were 31% higher than from March to May 2019.

**Fig 3:**
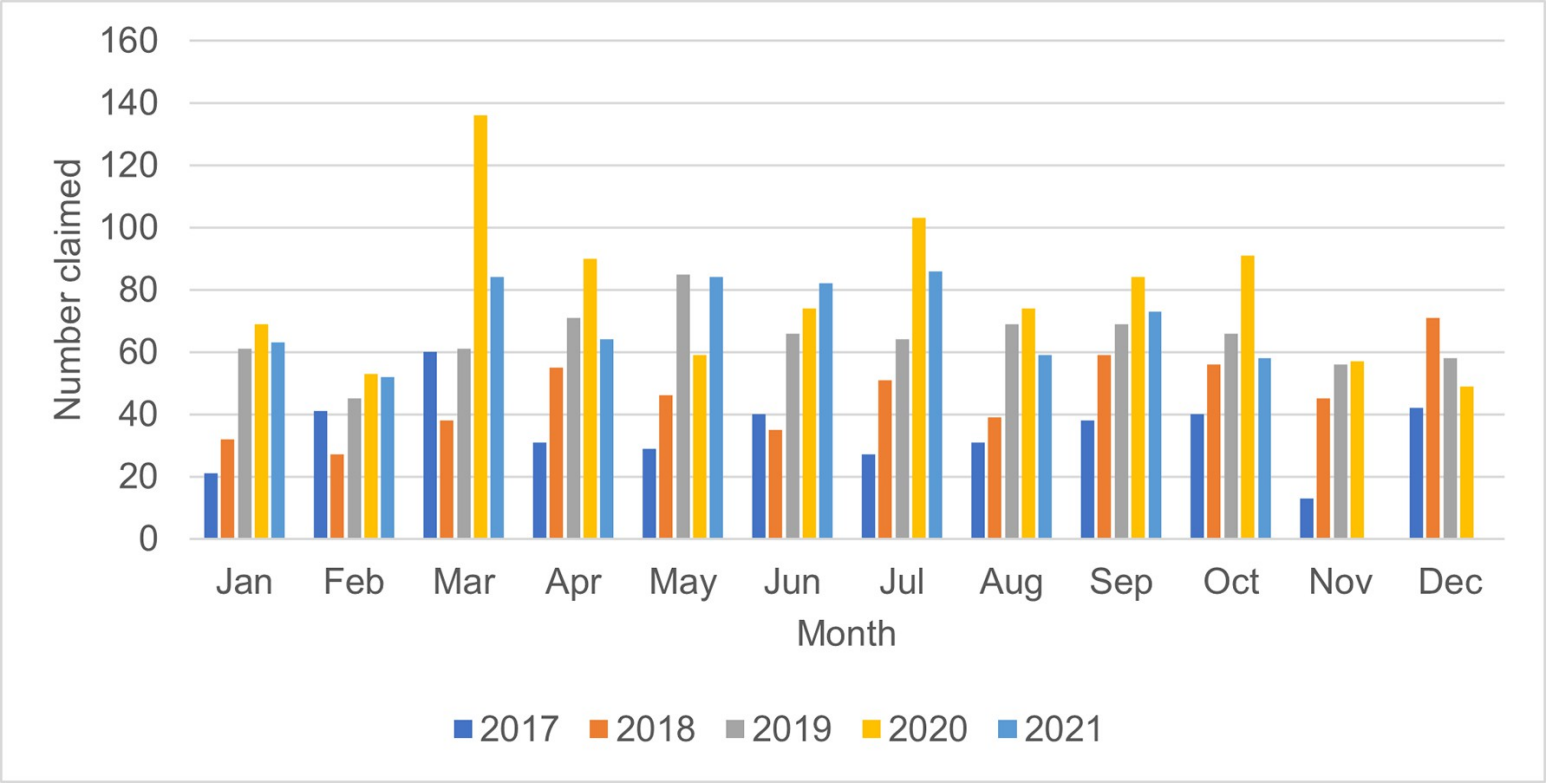
Uptake of maternal pertussis vaccination in pharmacy January 2017 to October 2021 by month of administration.

Subsequent outbreaks and lockdowns or increasing alert levels were not associated with the same increase in pharmacy MPV uptake as the first outbreak and lockdown. The August 2020 community outbreak in another region (Auckland) and nationwide increase in alert levels to level 2 did not appear to be associated with increased MPV uptake in Waikato pharmacies (Fig 3). The higher MPV uptake in July 2020 was not related to any additional COVID-19 activity. The delta outbreak leading to community cases of COVID-19 and the nationwide level 4 lockdown starting August 18 2021, were not associated with any increase in MPV uptake in pharmacy over the same period in 2019 or 2020. The peak administration of COVID-19 vaccinations in New Zealand took place in September and October 2021.

### Qualitative results

Eighteen consumers, 12 pharmacists, and 11 midwives were interviewed. Most interviews took 25 to 30 minutes (range 7–52 minutes). One participant declined recording with notes taken instead.

Consumers were aged 18–37 years, and identified as Māori (n = 9), NZ European or European (n = 7), Chinese (n = 1) and Cook Island Maori (Pasifika, n = 1). Eight consumers were pregnant at the time of interview; one was 22 weeks’ gestation and seven were 31–39 weeks’ gestation. Nine had infants aged five weeks to five months of age; one had an infant 12 months of age. All consumers used a midwife. Thirteen women first saw their midwife before or at 10 weeks’ gestation, two presented at 12–15 weeks’ gestation and three at 25–27 weeks’ gestation. Three women had received no vaccinations, five received both maternal vaccinations, one woman planned to receive both maternal vaccinations, eight received MVP only and one received maternal influenza vaccination only.

Pharmacists comprised 10 who were trained vaccinators in a community pharmacy providing vaccinations and two (one of whom was a trained vaccinator) who worked in a community pharmacy not providing vaccinations. Five of the pharmacists were pharmacy owners. Identified ethnicities were European (n = 6), Chinese or Asian (n = 3), Māori (n = 1), Fiji Indian (n = 1) and Middle Eastern (n = 1). Four had 1–4 years’ experience, five had 9–18 years’ experience and three had 30–40 years’ experience. Eight identified as female and four as male.

Ten of the midwives worked as Lead Maternity Carers who work independently in the community (n = 9) and one worked at a hospital. They identified as Māori or part-Māori (n = 5), NZ European or European (n = 5) and Asian (n = 1).

When asked about influences on their maternal vaccination decisions, most consumers spoke about HCPs (often midwives and/or pharmacists) informing them about one or both maternal vaccinations. While some participants appeared well-informed about maternal vaccinations, others had no awareness. In some cases, their only awareness came from the pharmacist mentioning maternal vaccinations:

“*To be quite honest I didn’t know anything before I got the whooping cough one and the flu jab in pregnancy [after the pharmacist raised it] and I actually thought you couldn’t get anything while you were pregnant until I did get that done and I was actually quite happy that I got it done.” Consumer 2*

Sometimes the HCP raising awareness did not result in vaccination, with Consumer 1 reporting having neither MPV nor maternal influenza vaccination, despite being advised to have maternal vaccinations by her midwife, general practitioner and pharmacy, but said that, in addition to being concerned about needles:

“*I guess I’ve never really had it explained to me though what the side effect or the bad effect could be if I didn’t.” Consumer 1*

A pharmacist indicated how further discussion was sometimes useful after another HCP had raised maternal vaccinations:

“*… they just need reassurance. So I’ve turned so many around when I’ve said ‘oh actually it’s recommended’ and I show them the posters because I’ve got two of them in my consult room and they go ‘oh okay that’s fine because my midwife has suggested it but I’m still a bit…’” Pharmacist 10*

When asked about what resources they had seen, most consumers had seen promotional material somewhere, e.g. posters and/or pamphlets at the general practice, midwife practices, pharmacies or in the hospital. However, the key reason for having a maternal vaccination tended to be influence from their HCP, primarily midwife, but also pharmacists or general practice staff.

“*Oh I saw posters everywhere. At the birth centre, again at the GP [general practitioner] and stuff like that. I was given information. Yeah there was information everywhere. Often it would be on the TV at the doctor’s as well…. it didn’t influence me. I think it was just because I’d heard first hand from my midwife, who I trust, that there was a rise in it in the Waikato and these were the reasons why people were potentially looking at it this time round so it wasn’t to do with any pamphlets or anything. It didn’t push it for me at all.” Consumer 7*

“*To be honest with you it just didn’t register*. *I saw [the MPV poster] and went ‘oh that’s a great idea’ [but then forgot about it]*.*” Consumer 10*

*“I don’t really look at pamphlets*, *but you know if I did see one I would probably grab it*.*” Consumer 6*

A midwife was unaware of Facebook posts on maternal vaccines and did not think her clients were influenced by it, but did note that a poster displayed in their waiting room helped:

“*I’ve never seen it [Facebook posts] and I’ve not heard about it. And no one’s come in and said “I’ve seen it on, I need to have that done”. I’ve not had any feedback to say “I’ve seen it on Facebook” or whatever “I need to have it done”. I’ve had women waiting in the waiting room [see the poster] and say “Oh I need to get that done soon too.” Midwife 1*

However, two other HCPs thought that people did not read or even look at posters, a pharmacist thought this because of low literacy in their population. A few HCPs suggested the same for pamphlets:

“*When it comes to the brochures, I used to give out a lot more than I do now but I’ve cut back a bit because you sort of realise that verbal works a lot better for most women than just handing them a brochure.” Midwife 6*

Social media and websites were not raised often by consumer participants. When discussed, social media appeared to be viewed with scepticism and was not particularly influential. A participant would disregard it because she would expect information from her midwife or wherever she would get the vaccination administered. Another noted that if seen on social media she would look into it more. Pharmacists noted some effect of Facebook posts provided by the study team:

“*…we shared that on the Facebook page and there are a few people that we saw that commented like that they tagged other people in it. So I assume they were saying “hey you’re pregnant, look at this” kind of thing.” Pharmacist 1*

Considering which components of the promotional material used appeared to have the most impact on uptake in the pharmacy, pharmacists reported that they most appreciated the phone calls encouraging pharmacists to talk to their midwives about having free maternal vaccinations in pharmacy, and for pharmacy staff to proactively discuss maternal vaccinations with pregnant people, including using prescriptions from midwives, e.g., for folic acid and iodine tablets (antenatal supplements recommended and funded on prescription), as a prompt.

Some midwives reported pharmacists contacting them about having free maternal vaccinations including pertussis. Several midwives reported discovering pharmacy provided maternal vaccinations from a regional newsletter for midwives, and two mentioned finding out in a webinar or course. Two midwives heard the pharmacy did it because one or more of their clients had their pertussis vaccination at the pharmacy:

“I’ve *had a whole tonne of women tell me they’ve been to the pharmacy and had it done so I think what we’ve seen happen, even in a short time, is one person does it who tells ten other people who tells ten other people and all of a sudden the fact that you can get it at Huntly West pharmacy has in the last two months gone viral, almost.” Midwife 6*

Although a list was sent to all midwives stating which pharmacies provided vaccinations across Waikato, and information that these were free in pharmacy, several midwife participants did not know pharmacy could provide maternal vaccinations, and some were unaware which pharmacies provided maternal vaccinations, or if they were free.

“*I don’t know which [pharmacies] provide them so I just tell them go to their GP [general practitioner].” Midwife 2 who reported 2 pharmacists had told her their pharmacies provided maternal vaccinations*

“*[I learnt pharmacists provide maternal vaccinations] at a midwife information thing…*. *But… I don’t use it a lot…*. *I don’t know if there was a fee attached to the chemist and I haven’t got all of that*.*” Midwife 7*

Some pharmacists reported that pregnant people had been suggested to go to the pharmacy by their midwife, sometimes this may have been prompted by the pharmacist’s discussion with the midwife.

“*I made a point of asking [people getting maternal vaccinations] and … 90% of them at least said their midwife told them they could get it in the pharmacy and just come in whenever because they didn’t need an appointment…” Pharmacist 3 who had not contacted local midwives about their service*

“*… regarding the whooping cough vaccination we also give the leaflets to midwives and ask them if they could tell the patients to come in here as well and most of them are happy to do so*, *so then we do some patients that say “my midwife told me this and that” so they come here to get it*.*” Pharmacist 7*

Some pharmacists reported more influence from the promotional activity than the other HCPs or consumers. When conducting interviews, the pharmacies that provided vaccinations usually had MPV posters on display, and sometimes the Fact Sheet with key points was visible on the dispensary wall, or the pharmacist said it was up in the staff room. However, posters were removed in some pharmacies when other posters needed to be put up, some pharmacies lacked room for posters, and most were unsure if the posters had helped in their pharmacy. Social media was used by a minority of pharmacists.

“*I think the main big way that [the Fact Sheet] did help was having better confidence and talking to the women when they came in so we could actually give them the big spiel about why they should do it and why their partners should do it and how it protects the baby after birth.… before it was a bit like ‘this is a good thing to do’ whereas now we can go into a bit more depth.” Pharmacist 3*

“*…we shared that on the Facebook page …*.*people were tagging other people in it*. *So it must have had some impact*.*” Pharmacist 1*

Several pharmacists spoke about the importance of other staff members in helping identify people who were pregnant and may need a maternal vaccination. Some pharmacies trained their staff, one finding the maternal vaccination quiz for pharmacy staff effective in supporting this.

“*… sometimes with the team members it’s a little thing too and a pressie card, one of my trainee techs won one of those and she was over the moon. Next time when we had that 20 people did the quiz…. if the team knows about the service then that’s easier because it can start with general conversations.” Pharmacist 10*

“*I think the techs that I have are pretty good*, *they’re pretty good at speaking to patients about the availability of being vaccinated here” Pharmacist 1*

Pharmacist proactivity with the promotional materials and maternal vaccinations varied (reported in Table 1). Two pharmacies which did not offer vaccinations had no posters on display, and the pharmacists from those pharmacies reported not telling pregnant people about maternal vaccinations. One pharmacy providing vaccinations was particularly active:

“*So we’ve been putting up posters on our windows and also a poster right outside this consultation room and in the consultation room as well and we also shared a picture of the posters on Facebook page and we did many clips on Facebook just to promote people to come in and we also hand out leaflets to people around say flu season. We put it in a bag so they take it home and read it or we tell them as we give out a prescription or we sell product. And … we also give [MVP] leaflets to midwives…. And also we have this focus of the month thing [and one month we did] whooping cough vaccination…. we tried to bring it up in each meeting that month…. To tell the staff to tell the customers… I think that was how we got nine [vaccinations] that month.” Pharmacist 7*

**Table 1:** Perceptions of promotional material by element and type of participant and uptake of promotional materials.

MPV Posters • Consumers ○ Some participants saw these in pharmacy, but generally their decisions about maternal vaccinations did not appear to be affected by them. ○ One person saw a MPV poster at their antenatal class. • Pharmacists ○ Most vaccinating pharmacies displayed these. ○ One participant reported taking some posters to a birth centre for display there to help raise awareness of MPV and free availability in pharmacy, but was unsure whether they were displayed or not ○ Some pharmacists noted that having the MPV posters up in their pharmacy reminded them to recommend MPV to pregnant people. • Midwives ○ Midwives were offered MPV posters in an email communication but very few responded to have them sent out
Facebook posts about maternal vaccinations from the study team, including videos • Participants eligible for maternal vaccinations ○ Most participants were unaware of the Facebook activity for this project ○ Most participants indicated a distrust of social media for vaccination information ○ However, some participants recommended social media be used to promote MPV ○ Where participants were shown the video clips used in Facebook, they had different clips which they preferred, indicating the need for different messaging to appeal to different people. • Pharmacists ○ Some pharmacists shared the links with social media created for this project on their Facebook pages. However, their social media activity tended to be managed by front-of-store not dispensary staff. ○ One pharmacy videoed their staff wearing MPV t-shirts with posters on display in the frame and put this on their Facebook page to promote the service. ○ Pharmacists noted it was common if doing vaccination Facebook posts generally to get negative comments from some people. • Midwives ○ Midwives reported seeing no Facebook activity for maternal vaccinations.
Video of paediatrician’s information on maternal vaccinations for HCPs sent by email with a link to the video to pharmacists and midwives • No pharmacist or midwife participants had watched these • The video viewership numbers were very low (under 30)
Emails developed by the study team with information about maternal vaccinations–sent directly from the study team to all pharmacists and via the College of Midwives to midwives in the region • Pharmacists ○ Most pharmacist participants reported remembering these and finding them helpful • Midwives ○ Some midwife participants recalled receiving these emails ○ They found the emails useful for awareness about MPV funding in pharmacy ○ Despite reading these emails which included a list of pharmacies providing maternal vaccinations, some midwives were not sure which pharmacies provided maternal vaccinations
Pharmacists contacted midwives to tell them they administered funded maternal vaccination as suggested by the project team • Pharmacists ○ Some vaccinating pharmacists acted on this suggestion and found it helpful in people coming to get maternal vaccinations from them • Midwives ○ Some midwives recalled receiving a phone call from the pharmacist about maternal vaccinations, and considered it helpful ○ Despite the phone call, some midwives forgot which pharmacies provided vaccinations so recommended going to the medical practice without mentioning pharmacy availability
Fact Sheet on maternal vaccinations and staff quiz sent to pharmacists • Most pharmacist participants found this useful. • The two pharmacists at non-vaccinating pharmacies had not noticed these elements • The staff quiz was used in some pharmacies and reported to increase staff knowledge about maternal vaccinations which helped staff identify people who were pregnant for discussion about maternal vaccination.
T-shirts with MPV messaging • About half of the vaccinating pharmacies requested these t-shirts for staff • One pharmacist considered them useful for starting conversations • Another pharmacist thought they had not raised awareness among pregnant people. • T-shirts were worn sporadically, but one used them for their staff in a short video for a Facebook post.

One pharmacist reported a pharmacy policy of phoning pregnant people to remind them to get their MPV vaccine when they reached the appropriate gestation.

#### Recommendations for promotion

Consumer participants wanted awareness raised further through proactivity from the midwife, pharmacy and general practice, distributing flyers, online information, Facebook, information at antenatal classes and videos of babies with whooping cough to understand the effects of it. A minority of women suggested dissemination of information was already sufficient. One consumer participant when asked how pregnant people should be informed that is it important to get these vaccinations in pregnancy replied:

“*I guess, like, the GP, like when you first find out that you’re pregnant, the GP could do it or the ultrasound people or even the midwife…. [the pharmacy] could give you a pamphlet… when you pick up a prescription or something, just to remind you.” Consumer 15*

Consumer 1 when asked this same question suggested extra support/funding for pharmacies and midwives to *“…push it a bit further… [because they] are places that new mothers or pregnant women … feel kind of kind of comfortable and secure [and] already have the relationships built with the mothers*, *rather than trying to start something new*.*”*

Recommendations from midwives were highly variable. One wanted no more information *“there is a lot of posters and paperwork and we don’t need more of that*.*”* A midwife whose clientele often had difficulty communicating in English wanted information to be available in multiple languages. Another midwife suggested providing more information about maternal vaccinations to midwives, including reassurance regarding safety. Other suggestions from midwives included texts to women, a billboard, incentive vouchers for the women (also recommended by one pharmacist) and reminders from general practice following their one funded antenatal visit there.

Pharmacists noted the importance of education for pharmacy staff, and sometimes suggested more of that. Pharmacists were also positive about having technicians in the pharmacy able to do vaccinations (at this time technicians could not vaccinate). One pharmacist suggested a sticker the pharmacy could put on the folic acid bottle when dispensed to remind the pharmacist to discuss vaccinations when dispensing folic acid to pregnant people. Another suggested sending reminders to pregnant people near their gestation when MPV could be given.

## Discussion

This study included data for MPV vaccination administration in pharmacy comprising 18 months of funded vaccination before the promotion, a six-month promotional period, and then three years of data following the promotional period, including during the COVID-19 pandemic and lockdowns. The findings suggest promotion improved uptake of MPV from pharmacies considerably, with further growth evident over the 18 months after the promotion campaigned was finished. The COVID-19 pandemic was initially associated with an increase in MPV uptake in pharmacies at a time of the first COVID-19 outbreak and strict lockdown. However MPV uptake in pharmacy was lower over the subsequent 12 month period.

Women were largely influenced to have MVP by discussion with HCPs. Although some saw pamphlets and posters advising of the importance of MVP and free availability at the pharmacy and general practice, participants usually reported the HCP’s influence to be greater.

Pharmacists from pharmacies delivering vaccinations welcomed the promotion from the study team, particularly the telephone calls suggesting discussing maternal vaccinations with people who were pregnant, including when dispensing a prescriptions for supplements used antenatally. This reportedly stimulated proactivity in some pharmacies and increased uptake. Some pharmacists also advised midwives of maternal vaccinations being provided at their pharmacy. Midwives appreciated the information from pharmacists and emails about free maternal vaccinations from the pharmacy, when they remembered receiving it. Encouraging pharmacists by phone and email to advise their local midwives that they provide free maternal vaccinations appeared helpful, although some pharmacists did not act on this suggestion. Information emailed to midwives about pharmacies providing maternal vaccinations was sometimes found useful, although some midwives were informed by other sources. Despite this, some midwives remained insufficiently informed about the availability of funded maternal pertussis vaccination in pharmacy. Some midwives were unsure which pharmacies provided the service, and this may improve over time as increasing numbers of pharmacies offer vaccinations.

HCPs are an important influence on vaccination during pregnancy [12,22,31], so understanding what effect promotion had in increasing proactivity in pharmacy, and which elements were useful is important.

There was some uncertainty about how effective posters, pamphlets, t-shirts and social media had been, although such resource gave pharmacies a visual reminder of maternal vaccinations in the pharmacy and supported them in their attempts to promote maternal vaccinations. Others have found that educational strategies with pregnant people have not been particularly effective while work with health care providers can increase uptake [23].

The initial MPV uptake increase before and during the first lockdown may have occurred if pregnant people switched from general practice to pharmacy to avoid general practices in case of being exposed to COVID-19 there, or if concerned that general practices were closed.

The later decline in MPV in pharmacy might reflect pharmacy burnout or different consumer behaviour e.g. pregnant people not attending the pharmacy for prescriptions in person or lower prioritisation of vaccinations other than COVID-19. In 2020, extended periods of lockdowns increased the workload in community pharmacies [32], potentially causing burnout or reducing the focus on maternal vaccinations. A survey of New Zealand pharmacists in March to May 2021 found high pressure of work and high psychological stress for pharmacists [33], but this was considered long-standing rather than specific to that time.

Vaccination rates in children in New Zealand have also dropped during COVID-19 times [34]. A study conducted in the United States found reduced uptake of maternal influenza and pertussis vaccinations during COVID-19 times [34], and a study conducted in the United Kingdom showed barriers created from COVID-19 prevented maternal vaccinations. However, our data excludes general practice, so it is possible that pregnant people continued to get vaccinated, but more used general practice rather than pharmacy at this time. During the later COVID-19 period there were no lockdowns in Waikato and only one case of COVID-19 in Waikato, so pregnant people should have been unconcerned about going to general practice or pharmacy to have a vaccination.

The increase in vaccinations over time after the promotion ceased may reflect increased confidence as the pharmacists and pharmacy staff discussed it with consumers, and increased word-of-mouth with pregnant people and midwives. However, the effect of promotion might wane over time, and further work may be required to boost uptake again.

While we found an increase in uptake in pharmacy, MPV rates remain low in New Zealand, particularly in groups disproportionately affected by pertussis infection [35]. Therefore, further work is needed to optimise the promotion, such as electronic reminders on computers with prescriptions of medicines typically used in antenatal care (i.e. folic acid and iodine), as has been found effective with other health care providers [23]. While provision of maternal vaccinations by antenatal providers is likely to be more effective in increasing uptake [23], given time constraints for midwives even to discuss maternal vaccinations [22,36], and logistical issues [22], such provision is unlikely to become widespread in New Zealand in the near future, so existing access points need to be optimised.

### Strengths and limitations

The study included five years of data, a long period for a pharmacy vaccination study, and included an 18 months period pre-promotion, then uptake after promotion, again for an extended period, enabling an opportunity to understand the effect of promotion. This study also included qualitative data on promotion to indicate possible reasons for the quantitative findings.

We relied on pharmacy claims data which was not verified to ensure the claims were only for pregnant people. However, pharmacies were not funded for pertussis vaccination in other groups, and can be audited, so data is not expected to include pertussis vaccinations outside of pregnancy. Pharmacy claims were manual, allowing the possibility of omissions or late submitting claims. Data were extracted on 8 December 2021 to allow for late submissions.

For the qualitative interviews, discussion about promotional material was secondary to barriers and enablers to maternal vaccination and therefore this discussion was not in depth. Participants were reliant on their memory and for the consumer participants, their pregnancy may have been up to one year before the interview, which could affect recall about what promotional material they saw and its effect on their decision to have a vaccine or not.

The number of pharmacies providing vaccinations was only sought once, in April 2018.

Our social media was limited, and a different campaign may have increased interest and effect.

### Further research

There is a need to better understand how to optimise promotion of maternal vaccinations to HCPs and pregnant people to improve uptake further.

## Conclusion

Widening funded access of MPV to pharmacy needs to be accompanied by promotional activity to pharmacy and midwives to increase the benefit of this access. Promotion appeared to have a sustained effect on uptake of MPV beyond the promotion period but may need to be repeated periodically. Verbal discussions about MPV increased proactivity of pharmacists, and their discussions with pregnant people are important in aiding MPV uptake.

## Supporting information

S1 Table Covid timeline in New Zealand. (DOCX)

S1 File Qualitative question guides. (DOCX)
