# Supplementary material for: Promotion and COVID-19 lockdown increase uptake of funded maternal pertussis vaccination in pharmacy: A mixed methods study
Source: PLoS One. 2024 Aug 29;19(8):e0307971. doi: 10.1371/journal.pone.0307971 (PMC11361654; doi:10.1371/journal.pone.0307971)
Supplement: S1 Table — (DOCX) [file pone.0307971.s001.docx]

**S1 Table. Covid timeline in New Zealand**

| Period | Activity |
| --- | --- |
| 28 Feb 2020 | First confirmed case of COVID-19 in New Zealand |
| 14 Mar 2020 | New cases in NZ announced daily |
| 23 Mar 2020 | First cases of community transmission |
| 26 Mar 2020 | First day of nationwide lockdown, level 4 (strictest lockdown) |
| 2-5 Apr 2020 | Peak of the first outbreak |
| 28 Apr 2020 | First day of nationwide lockdown, level 3 |
| 15 May 2020 | First day of Alert level 2, nationwide |
| 29 May 2020 | No new cases of COVID-19 in the community for seven days |
| 10 Jun 2020 | First day of Alert level 1, nationwide (minimal restrictions) |
| 12 Aug 2020 | Return to Alert level 3 in Auckland and Alert level 2 elsewhere following community cases in Auckland |
| 31 Aug 2020 | Auckland to level 2.5, elsewhere remains level 2 |
| 22 Sep 2020 | First day that non-Auckland regions go to level 1, Auckland remains level 2.5 |
| 24 Sep 2020 | First day that Auckland moves to level 2 |
| 8 Oct 2020 | Auckland returns to level 1 |
| Nov 2020 | A very low level of community transmission but no changes to alert levels |
| 24 Jan 2021 | Single community case in Northland, no lockdown |
| 6 Feb 2021 | One new community case (after leaving managed isolation) reported in Waikato |
| 14 Feb 2021 | Three new cases in the community in Auckland |
| 15 Feb 2021 | First day of Auckland at alert level 3, elsewhere remains level 1 |
| 18 Feb 2021 | First day of Auckland at alert level 2, elsewhere remains level 1 |
| 20 Feb 2021 | First COVID-19 vaccine administrations in New Zealand starting with frontline workers |
| 27 Feb 2021 | Auckland to Alert level 3, elsewhere to level 2 following concerns about increased community exposure |
| 7 Mar 2021 | Auckland to Alert level 2, elsewhere to Alert level 1 |
| 12 Mar 2021 | Auckland to Alert level 1 |
| 27 July 2021 | COVID-19 vaccinations available for people in the community aged ≥60 years |
| 17 August 2021* | Delta outbreak starts, alert level 4 lockdown for the country |
| 31 August 2021 | Alert level 3 for all of New Zealand (including Waikato) except Auckland and Northland |
| 7 Sep 2021 | Revised alert level 2 for all of New Zealand (including Waikato) except Auckland |
| 20 Sep 2021 | Northern Hauraki (northern Waikato) alert level 4 |
| 22 Sep 2021 | Northern Hauraki (northern Waikato) alert level 3 |
| 25 Sep 2021 | Northern Hauraki (northern Waikato) alert level 2 as for the whole country. |
| 3 Oct 2021 | Community cases in Waikato see localised areas move to alert level 3 |
| 4 Oct 2021 | NZ moves from elimination to suppression strategy following high levels of vaccination across all areas |
| 15 Oct 2021 | Unlinked cases of COVID-19 reach 100 suggesting the Delta virus is uncontained |
| 27 Oct 2021 | Alert levels ease to alert level 3 step 1 for Waikato |

<https://www.policycommons.ac.nz/covid-19-policy-resources/covid-19-timeline/>

* <https://covid19.govt.nz/about-our-covid-19-response/history-of-the-covid-19-alert-system/> information used, differing from the site otherwise used.

Level 4 = strictest lockdown, work and learn from home, stay home. No gatherings, no mixing households. Essential businesses only allowed to be open (e.g., supermarkets, pharmacies, petrol stations).

Level 3 = Can travel for work, school, low-risk recreational activities, and travel between regions limited. Can mix to one other household. Work and learn from home if possible. Gatherings of up to 10 people allowed for specific events (e.g. weddings, funerals). Only certain businesses can serve customers in-person.

Level 2 = Businesses, schools, early learning services, tertiary education providers and public facilities open, maximum 100 people in a gathering. Physical distancing of event facilities. Masks required in many situations. No travel restrictions.

Level 1 = Record keeping of where you have been. No restrictions on personal movement or gatherings. Face masks on public transport and airplanes.
