# Supplementary material for: Promotion and COVID-19 lockdown increase uptake of funded maternal pertussis vaccination in pharmacy: A mixed methods study
Source: PLoS One. 2024 Aug 29;19(8):e0307971. doi: 10.1371/journal.pone.0307971 (PMC11361654; doi:10.1371/journal.pone.0307971)
Supplement: S1 File — (DOCX) [file pone.0307971.s002.docx]

**Qualitative Question Guides**

**Question Guide for people who are pregnant or who have delivered a baby in the last 12 months**

**NB Early interviews may result in new areas being probed. A conversational approach means questions will vary according to how the person answers and the flow of the interview.**

A conversational approach will be used in which the following questions/topics will be covered where possible:

Introduction and thanks for being available for the interview.

Demographics: age, ethnicity, number of children, living arrangements [ask how many people usually live in the house where they live], where they live (suburb and town, etc). How many weeks pregnant they are currently, or how old their baby is (if post-delivery).

1. Which LMC was used for their pregnancy – midwife in the community, midwife at the hospital, GP, obstetrician/gynaecologist. How many months pregnant were they when the LMC was first seen.
2. What do you know about whooping cough in babies?
3. What do you know about influenza in pregnancy? [if necessary probe: did they know that influenza is more dangerous in pregnancy for the Mum, e.g. she will have a higher risk of hospitalisation.
4. Do you know if you had your childhood vaccinations – e.g. vaccines at school or at the doctors?
5. What do you know about vaccines being given in pregnancy? Probe: what did you hear or read? [important] Where did you get your information? [probe on whooping cough/pertussis and influenza]
6. What do you think about the idea of vaccines in pregnancy?
7. Did you have any vaccinations given to you during your pregnancy?

**If vaccinated:**

1. What vaccination/s were given? [prompt if necessary, influenza, pertussis]
2. Where did you get the vaccinations? [GP, practice nurse, midwife, hospital, pharmacist, work, other] Why did you go there for it?
3. When did you have the vaccinations? [how many weeks were you - influenza, pertussis]
4. Tell me about your decision to get a vaccination [probe, why did you, who influenced you, how did you know about it? Did you look up information on the internet or anywhere else?]
5. Who did you discuss it with?
6. Where did you find out about it? [probe: hospital, midwife, GP, pharmacy, poster, information sheet, family, friends, antenatal class, hapū wananga, Facebook, other? Probe also how: e.g. was it suggested, did they see written material, a video?]
7. If written information or social media or other media was used, where did you see or hear this? [probe also for how much it influenced their decision, what they thought about it]
8. What was the most important thing that helped you decide to have the vaccination?
9. Did you have any concerns about the vaccine? [if so, what were they, where did they come from (e.g. friends, health professionals, websites), and how were they overcome?]
10. What was your experience of getting the vaccine?
11. Probe about pertussis primarily but also influenza vaccination.

**If not vaccinated with any vaccination during pregnancy, or if one vaccine was given but not the other:**

Tell me about that. [probe: what were the reasons why they had not had a vaccination?]

Had anyone discussed the vaccine/s with you, [probe midwife, GP, nurse in the GP, nurse at the hospital, pharmacy staff, friends, family, other?] if so who discussed it with you? What did they say, and how did that influence your decision? What did you think of that discussion? Did you get enough information about the vaccination?

Was there anything that made it difficult for you to get the vaccine? [probe if necessary: expecting a cost, transport difficulties, booking an appointment, difficulty finding the time, pressure/persuasion from others not to be vaccinated, lack of child care]

Did you have any concerns about the vaccine? [if so, what were they? What was the most important concern? Where do you hear about that concern?, e.g. Facebook, friends, family]

Did you see any information about getting vaccinated during pregnancy [prompting: posters, Facebook, leaflets, anything else?] What did you think about these? How did it influence you (if at all)?

**For all:**

1. What do you think about vaccination generally?
2. Have you had your children vaccinated?
3. Do you know where pregnant women can get free vaccines for influenza and pertussis from? [prompt general practice, hospitals, pharmacies]
4. What do you think about the vaccination during pregnancy being available free from pharmacies?
5. Why would or wouldn’t you go to the pharmacy for a vaccination in pregnancy?
6. What could help other women like you to find out about the vaccination? [do posters help, Facebook posts or videos, recommendations from friends or family, pamphlets, a video in store, a reminder in the Bounty pack?]
7. What could make it easier for women like you to get vaccinated during pregnancy?

LMC = lead maternity carer

GP = general practitioner

**Question Guide – Healthcare providers**

**Probe as appropriate throughout on pertussis and/or influenza. Semi-structured, conversational approach, ensure key topics are covered but some questions can be missed if time is short and other areas explored if needed according to participant responses.**

**Introduction and thanks for being available for the interview.**

Demographics: Number of years practising as a [e.g. doctor]. Gender, ethnicity. Where do they practice? How long have they practised there? How many hours per week do they work?

What role if any do you have in vaccinations? [if necessary probe on the following do vaccinations, recommend vaccinations, provide information, do reminders for vaccinations by phone/mail?]

**For those administering vaccinations:**

How long have you administered vaccinations?

How many vaccinations would you do per week or per month [specify which]?

How many vaccinations of pregnant women would you have administered in the last month?

**For all:**

What do you think about the idea of giving vaccinations during pregnancy?

Tell me about your understanding of vaccination recommendations during pregnancy? [probe: pertussis and influenza if necessary, recommended timing, funding, and where they are available from in Waikato]

Where do you recommend women get their maternal vaccination? Why?

Why do you think some women do not get vaccinations during pregnancy?

What do you think helps women get vaccinations during pregnancy?

Are there any particular groups who you think are more likely to get vaccinations during pregnancy? If so, why are they?

Are there any particular groups who you think are less likely to get vaccinations during pregnancy? If so, why not?

What do you think needs to be done to increase the number of women getting vaccinations during pregnancy?

What (if anything) have you or your practice done to help women get vaccinated during pregnancy? [probe: pamphlets, posters, recommendations, telephone reminders, no wait vaccines]. How well do you think that is working?

Have you seen any messages for women around for getting the pertussis vaccine or the flu vaccine during pregnancy, seen anything on Facebook? There’s been Facebook promotion of maternal vaccinations as part of this.

**For providers of vaccines:**

How does it work for your practice/pharmacy/you personally to provide vaccinations to pregnant women?

What challenges do you/your practice experience with vaccines? [probe if necessary: staff availability, fridge space, out of stocks, insufficient funding, too busy]

How long does it take to get an appointment in [your practice/pharmacy, or in local providers, as applicable] for a vaccination? If appropriate: What days and times can people get vaccines?

What helps you provide maternal vaccinations in your practice/pharmacy?

**For midwives, doctors and practice nurses:**

Did you know that in the Waikato some pharmacies can administer funded pertussis and influenza vaccines during pregnancy?

How did you hear about it?

What do you think about that?

Have you had any communication from your local pharmacy about it?

What do you think about pharmacies providing these?

Have you ever recommended a woman go to a pharmacy for a maternal vaccination? If so, how often? Why or why not?

**For midwives**

A list of all the pharmacies and the locations and the opening times was sent from your regional representative from the College of Midwives so that you knew which pharmacies would do it. Do you recall getting that? How useful was it?

Did

**For pharmacy:**

Does your pharmacy provide vaccines or not? Why/why not?

What other barriers are there for pharmacy generally to providing the vaccinations? [if necessary probe stock, fridge, staff aspects, busyness, inadequate funding, accessing training, patients do not want it, no interest or think it is not appropriate to pharmacy, no consultation room, or consultation room used for other reasons, concern about liability, do not like vaccines, paperwork eg SOPs, set up costs, local GP concern]

What other enablers are there for pharmacy generally to providing the vaccinations? [probe: funding, help from pharmacy organisations, encouragement from other health providers or the DHB]

Which of your staff members know about the maternal vaccinations?

What do you think about pharmacy technicians being able to vaccinate?

**For pharmacies providing vaccines:**

How many pregnant women per week would be getting vaccinated at your pharmacy?

Tell me about how most pregnant women present for vaccinations [e.g. patient request, suggested in the pharmacy, get an idea of the proportion of each]

When you are talking about maternal vaccinations with women, how often are they aware about maternal vaccinations do you think, before you brought it up?

How often is it given on the spot versus by appointment?

What interaction did you have, if any, with your local general practice/s regarding the service? What was their response?

What interaction did you have, if any, with your local midwives regarding the service? What was their response?

What resources did you use with maternal vaccinations, posters, pamphlets, anything else?

What did you think of the promotional material and how useful was it? [probe: posters, phone calls and emails from the study team to the pharmacy, the fact sheet for pharmacy staff, staff quiz, Facebook]

Have you seen the social media posts on Facebook on maternal vaccinations [described], and if so, what did you think about these?

Look to see if the posters are on display in the pharmacy.

**For pharmacies not providing vaccines:**

What role, if any, do you have in vaccinations?

What are the barriers for pharmacy to provide vaccinations? [probe training, refrigeration, medical centre, time, liability]

What were your thought processes about whether your pharmacy would deliver vaccinations or not?

What do you remember of any communication about maternal vaccinations?

Look to see if the posters are on display in the pharmacy.
